# Impact of *TMPRSS2* Expression, Mutation Prognostics, and Small Molecule (CD, AD, TQ, and TQFL12) Inhibition on Pan-Cancer Tumors and Susceptibility to SARS-CoV-2

**DOI:** 10.3390/molecules27217413

**Published:** 2022-11-01

**Authors:** Jiewen Fu, Shuguang Liu, Qi Tan, Zhiying Liu, Jie Qian, Ting Li, Jiaman Du, Binghui Song, Dabing Li, Lianmei Zhang, Jiayue He, Kan Guo, Baixu Zhou, Hanchun Chen, Shangyi Fu, Xiaoyan Liu, Jingliang Cheng, Tao He, Junjiang Fu

**Affiliations:** 1 Key Laboratory of Epigenetics and Oncology, the Research Center for Preclinical Medicine, Southwest Medical University, Luzhou 646000, China; 2 Basic Medical School, Southwest Medical University, Luzhou 646000, China; 3 Department of Pathology, The Affiliated Huaian No. 1 People’s Hospital of Nanjing Medical University, Huai’an 223300, China; 4 Department of Gynecology and Obstetrics, Guangdong Women and Children Hospital, Guangzhou 511400, China; 5 Department of Biochemistry, School of Life Sciences, Central South University, Changsha 410013, China; 6 School of Medicine, Baylor College of Medicine, Houston, TX 77030, USA; 7 Institute for Cancer Medicine, Basic Medical School, Southwest Medical University, Luzhou 646000, China

**Keywords:** *TMPRSS2* gene, SARS-CoV-2, prostate adenocarcinoma, susceptibility, cordycepin (CD), adenosine (AD), thymoquinone (TQ), TQFL12

## Abstract

As a cellular protease, transmembrane serine protease 2 (TMPRSS2) plays roles in various physiological and pathological processes, including cancer and viral entry, such as severe acute respiratory syndrome coronavirus 2 (SARS-CoV-2). Herein, we conducted expression, mutation, and prognostic analyses for the *TMPRSS2* gene in pan-cancers as well as in COVID-19-infected lung tissues. The results indicate that TMPRSS2 expression was highest in prostate cancer. A high expression of TMPRSS2 was significantly associated with a short overall survival in breast invasive carcinoma (BRCA), sarcoma (SARC), and uveal melanoma (UVM), while a low expression of TMPRSS2 was significantly associated with a short overall survival in lung adenocarcinoma (LUAD), demonstrating TMPRSS2 roles in cancer patient susceptibility and severity. Additionally, TMPRSS2 expression in COVID-19-infected lung tissues was significantly reduced compared to healthy lung tissues, indicating that a low TMPRSS2 expression may result in COVID-19 severity and death. Importantly, TMPRSS2 mutation frequency was significantly higher in prostate adenocarcinoma (PRAD), and the mutant TMPRSS2 pan-cancer group was significantly associated with long overall, progression-free, disease-specific, and disease-free survival rates compared to the wild-type (WT) TMPRSS2 pan-cancer group, demonstrating loss of functional roles due to mutation. Cancer cell lines were treated with small molecules, including cordycepin (CD), adenosine (AD), thymoquinone (TQ), and TQFL12, to mediate TMPRSS2 expression. Notably, CD, AD, TQ, and TQFL12 inhibited TMPRSS2 expression in cancer cell lines, including the PC3 prostate cancer cell line, implying a therapeutic role for preventing COVID-19 in cancer patients. Together, these findings are the first to demonstrate that small molecules, such as CD, AD, TQ, and TQFL12, inhibit TMPRSS2 expression, providing novel therapeutic strategies for preventing COVID-19 and cancers.

## 1. Introduction

Transmembrane serine protease 2 (TMPRSS2, OMIM: 602060) encodes a 492 amino acid protein with a molecular weight of 53,859 Da, which contains a functional serine protease domain [1]. TMPRSS2 has been reported to be involved in various pathological and physiological processes [2,3,4]. Recurrent fusions at the *TMPRSS2* 5′ UTR (untranslated region) to ETV1 (ETS variant transcription factor 1) or ERG (ETS-related gene) lead to outlier expression and drive progression of prostate cancer [5].

Serine proteases proteolytically cleave and activate viral S proteins (spike glycoproteins), thereby facilitating fusion of the virus with the cell membrane. Two independent mechanisms are involved in human severe acute respiratory syndrome coronavirus (SARS-CoV) infection entering host cells: (1) proteolytic cleavage of angiotensin converting enzyme 2 (ACE2), which promotes viral entry; and (2) cleavage of coronavirus S protein, which activates itself. Proteolytic cleavage and activation of the S protein is required for infection of human coronavirus 229E (HCoV-229E) and human coronavirus EMC (HCoV-EMC), as well as the F0 fusion glycoprotein of Sendai virus (SeV), human metapneumovirus (HMPV), and human parainfluenza viruses (HPIVs) 1, 2, 3, 4a, and 4b.

As a cellular serine protease, TMPRSS2 is a genetic risk factor [6,7] as it facilitates viral entry, including HCoV-229E, HMPV, Middle East respiratory syndrome coronavirus (MERS-CoV), and SARS-CoV, through cleaving and activating viral envelope glycoproteins, thereby facilitating the fusion of virus with cell membranes, or through proteolytically cleaving of ACE2, which promotes viral uptake in a cathepsin L (CTSL)-independent manner [8,9,10,11].

Hoffmann et al. first demonstrated that the SARS-CoV-2 S protein is primed via the TMPRSS2 protease, indicating that TMPRSS2 inhibitors may block viral invasion [9,12], SARS-CoV-2 entry, and coronavirus disease 2019 (COVID-19) outcomes [13]. These findings suggest that the inhibition of TMPRSS2 expression may protect against cancer progress as well as SARS-CoV-2 invasion. Variants in the *TMPRSS2* gene seem to regulate TMPRSS2 expression and affect SARS-CoV-2 infection [14,15,16]. Gene mutations in cancer could cause gene expression, malignancy, prognostics, recurrence, and therapeutic resistance of patients. TMPRSS2 expression, mutation, and prognostics in pan-cancers are unclear. Some potential inhibitors of TMPRSS2 have been revealed to possibly defeat SARS-CoV-2 entry [17,18,19]. It is unknown whether small molecules, such as cordycepin (CD), adenosine (AD), thymoquinone (TQ), and TQFL12, inhibit TMPRSS2 expression.

Herein, we conducted expression, mutation, and prognostic analyses for the *TMPRSS2* gene in pan-cancers as well as in COVID-19-infected lung tissues. Cancer cell lines were treated with the small molecules CD, AD, TQ, and TQFL12 to inhibit TMPRSS2 expression in cancer cell lines.

## 2. Results 

### 2.1. TMPRSS2 Expressions and Prognostics in Pan-Cancers

**Figure 1:**
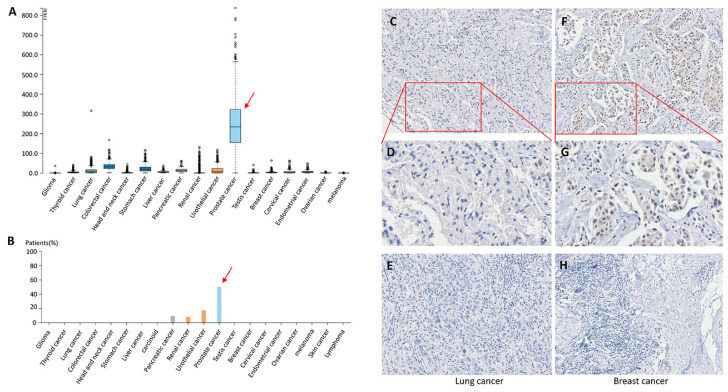
TMPRSS2 expression in pan-cancers. (**A**) *TMPRSS2* mRNA expression in pan-cancers. (**B**) TMPRSS2 protein expression in pan-cancers. (**C**,**D**) IHC of TMPRSS2 in lung cancer tissues. (**E**) IHC of TMPRSS2 in lung cancer tissues. (**F**,**G**) IHC of TMPRSS2 in breast cancer tissues. (**H**) IHC of TMPRSS2 in breast cancer tissues. (**D**,**G**) Enlarged images of (**C**,**F**), respectively.

Previous bioinformatics analysis of *TMPRSS2* expression profiles in 32 different tumor tissues has indicated that *TMPRSS2* is significantly upregulated in six cancer tissues, including prostate adenocarcinoma (PRAD), and significantly downregulated in six cancer tissues compared to corresponding normal tissues [20]. We further analyzed *TMPRSS2* mRNA expression in pan-cancers and found that *TMPRSS2* mRNA expression was highest in prostate cancer with 234.4 fragments per kilobase of exon model per million mapped fragments (FPKM) (Figure 1A). TMPRSS2 protein expression was highest in prostate cancer followed by urothelial cancer, renal cancer, and pancreatic cancer, and TMPRSS2 was expressed at low levels in lung cancer; TMPRSS2 expression was undetected in the remaining cancer tissue types, including breast cancer (Figure 1B). Moreover, immunohistochemistry (IHC) showed weak to moderate membranous and/or granular cytoplasmic immunoreactivity in lung and breast cancer tissues (Figure 1C–H).

**Figure 2:**
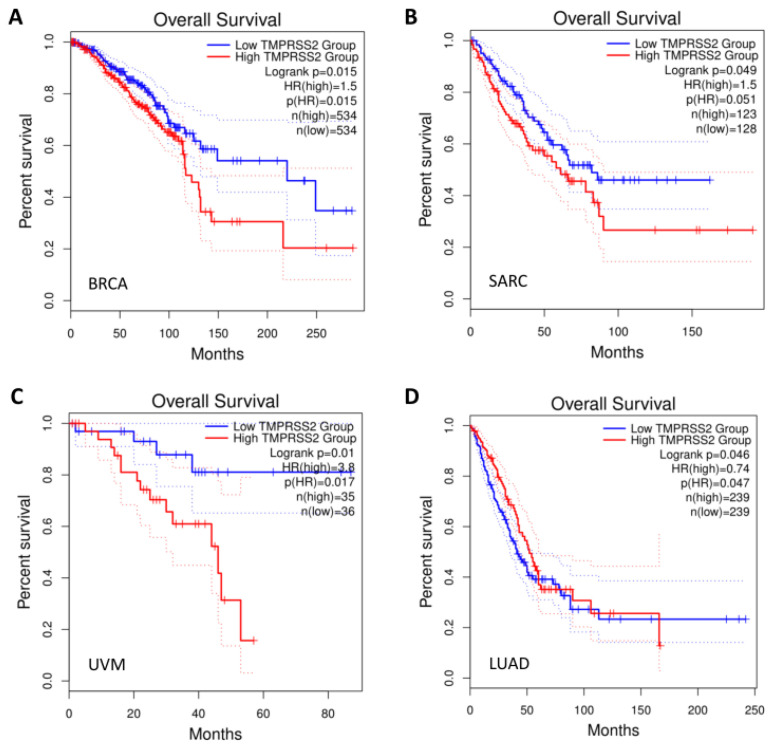
Overall survival of cancer patients according to TMPRSS2 expression. (**A**) BRCA. (**B**) SARC. (**C**) UVM. (**D**) LUAD. BRCA: breast invasive carcinoma; SARC: sarcoma; UVM: uveal melanoma; LUAD: lung adenocarcinoma.

We next conducted survival analysis in the pan-cancers. A high expression of TMPRSS2 was significantly associated with a short overall survival in breast invasive carcinoma (BRCA), sarcoma (SARC), and uveal melanoma (UVM), as well as with a long overall survival in lung adenocarcinoma (LUAD) (Figure 2A–D). 

### 2.2. TMPRSS2 Mutations and Prognostics in Pan-Cancers 

**Figure 3:**
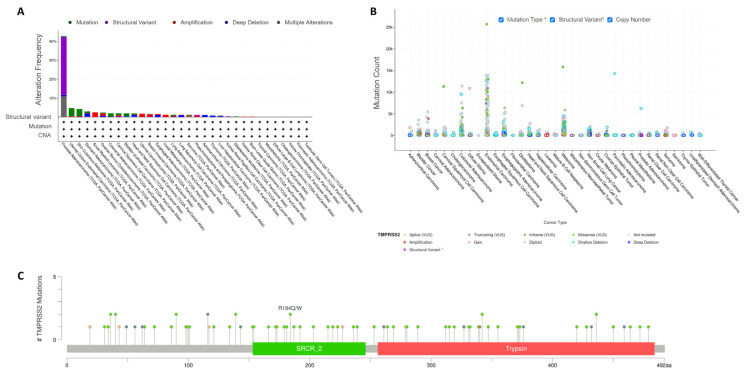
*TMPRSS2* mutation in pan-cancers. (**A**) Cancer type summary for *TMPRSS2* mutations, including 32 categories (cancer study) based on filtering. (**B**) Plots for *TMPRSS2* mutation types. (**C**) Overview of mutations in *TMPRSS2*. CNA: Copy number alterations (CNA) data.

Mutation analysis of the pan-cancers demonstrated that the mutation frequency was significantly high in PRAD (42.71%) and low in uterine corpus endometrial carcinoma (UCES) (4.73%), and 7 of the 32 types of cancer had no mutations (Figure 3A). The plots for the *TMPRSS2* mutation types are shown in Figure 3B, and an overview of mutations in *TMPRSS2* is shown in Figure 3C. Different types of *TMPRSS2* mutations were found, and missense mutations were the dominant mutation type. 

**Figure 4:**
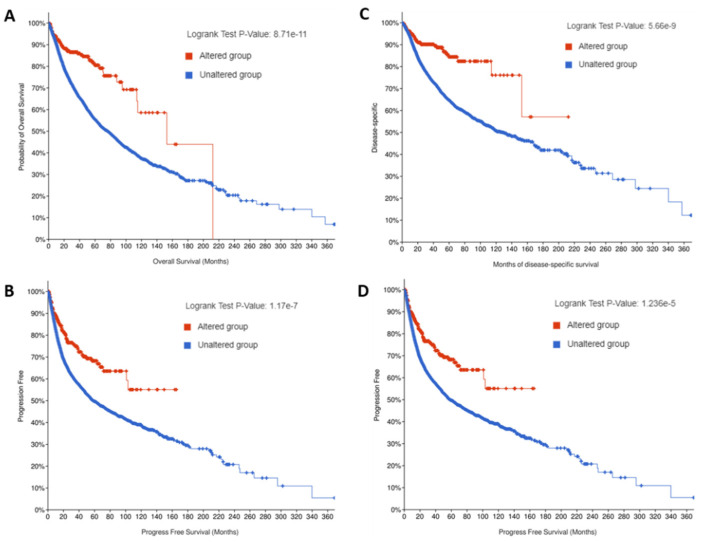
Prognostics for *TMPRSS2* mutation in pan-cancers. Data for overall (**A**), progression-free (**B**), disease-specific (**C**), and disease-free (**D**) survival in the mutated group (red line) and WT group (blue line). Unaltered group: wild-type (WT) group.

Further survival analysis was conducted with and without *TMPRSS2* mutations in the pan-cancers, which demonstrated that the mutated *TMPRSS2* group was significantly associated with long overall (Figure 4A), progression-free (Figure 4B), disease-specific (Figure 4C), and disease-free (Figure 4D) survival rates compared to the wild-type (WT) *TMPRSS2* group (Table 1). Thus, these findings indicate that *TMPRSS2* mutations may be a prognostic marker for long survival rates in pan-cancers.

### 2.3. CD, AD, TQ, and TQFL12 Inhibit TMPRSS2 Expression in Cancer Cell Lines 

**Figure 5:**
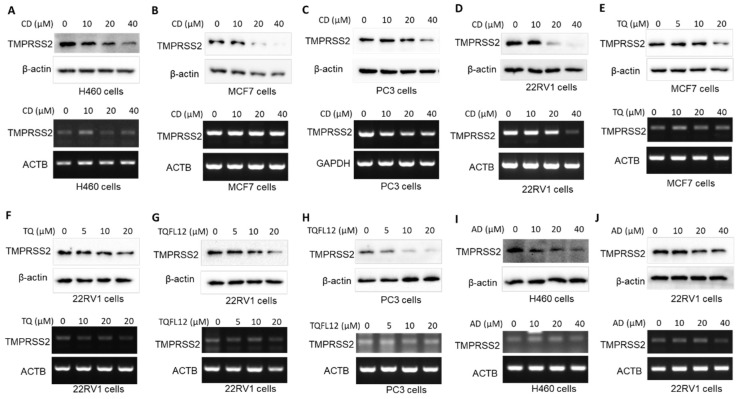
CD, TQ, TQFL12, and AD inhibit TMPRSS2 expression in various cancer cell lines. (**A**) CD inhibited TMPRSS2 expression in H460 cells. (**B**) CD inhibited TMPRSS2 expression in MCF7 cells. (**C**) CD inhibited TMPRSS2 expression in PC3 cells. (**D**) CD inhibited TMPRSS2 expression in 22RV1 cells. (**E**) TQ inhibited TMPRSS2 expression in MCF7 cells. (**F**) TQ inhibited TMPRSS2 expression in 22RV1 cells. (**G**) TQFL12 inhibited TMPRSS2 expression in 22RV1 cells. (**H**) TQFL12 inhibited TMPRSS2 expression in PC3 cells. (**I**) AD inhibited TMPRSS2 expression in H460 cells. (**J**) AD inhibited TMPRSS2 expression in 22RV1 cells.

Molecular docking analysis has predicted that small molecules have functional inhibitory effects on TMPRSS2 [21]. Thus, we investigated whether small molecules (CD, AD, TQ, and TQFL12) regulate TMPRSS2 expression. The results indicate that CD inhibited TMPRSS2 expression in H460 (Figure 5A), MCF7 (Figure 5B), PC3 (Figure 5C), and 22RV1 (Figure 5D) cells at the protein level in a dose-dependent manner. Moreover, TQ inhibited TMPRSS2 expression in MCF7 cells (Figure 5E) and 22RV1 cells (Figure 5F) at the protein level in a dose-dependent manner, and TQFL12 inhibited TMPRSS2 expression in 22RV1 (Figure 5G) and PC3 cells (Figure 5H) at the protein level in a dose-dependent manner. Further, AD inhibited TMPRSS2 expression in H460 (Figure 5I) and 22RV1 cells (Figure 5J) at the protein level in a dose-dependent manner. However, the small molecules did not significantly change *TMPRSS2* mRNA levels, except for CD treatment of 22RV1 cells. 

### 2.4. Treatment with CD Inhibits the Translation and Promotes the Degradation of TMPRSS2 Protein

**Figure 6:**
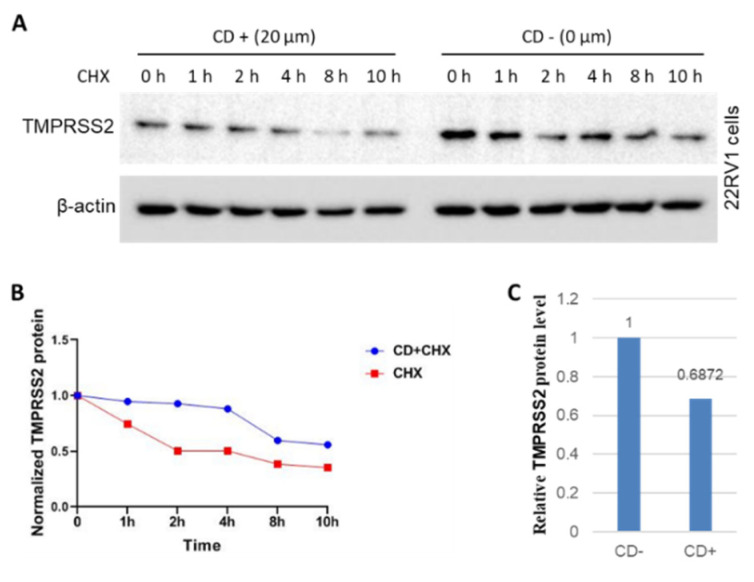
Treatment with CD inhibits the translation and promotes the degradation of TMPRSS2 in 22RV1 cancer cells. (**A**) TMPRSS2 protein stability after CHX treatment with or without CD treatment. The left panel shows CD treatment, and the right panel shows without CD treatment. (**B**) Quantitative results from A. The red line shows CHX treatment only, and the blue line shows CHX + CD treatment. Please note that the amount of TMPRSS2 protein in CD+ treatments remained lower over the entire period than that in CD−. (**C**) Quantitative results’ comparison of TMPRSS2 protein levels without and with CD treatments but without CHX treatments in Figure 6A. Left column shows without CD treatments (CD−), while right column shows with CD treatments (CD+). The final concentration of CHX was 40 µg/mL. h, hour (s) of CHX treatment. In the left panel of Figure 5A, all the lanes were added CD for 1 h prior adding CHX treatments.

We next investigated the protein stability of TMPRSS2 protein using cycloheximide (CHX) treatment in the presence or absence of CD treatment in 22RV1 cancer cells. The results showed that CD treatment increased the protein stability of TMPRSS2 compared to the control with an increase in the half-life from ~3 h to >8 h (Figure 6A,B). To further verify whether CD decreases the protein level of TMPRSS2, we quantitated TMPRSS2 protein levels and found that CD treatment decreased the TMPRSS2 protein levels by more than 30% (Figure 6C). Overall, these results indicate that CD treatment inhibits the translation and promotes the degradation of TMPRSS2 protein.

### 2.5. TMPRSS2 Expression in COVID-19-Infected Lungs and Control Lungs

**Figure 7:**
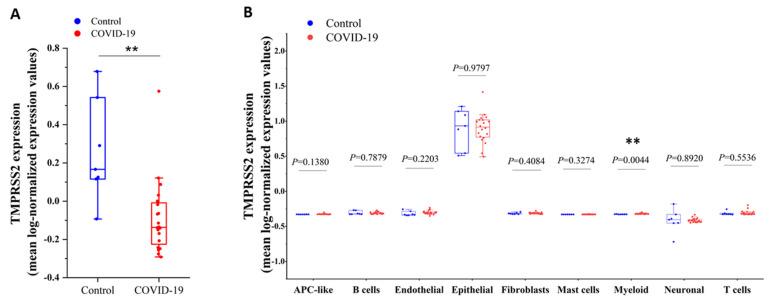
Expression of *TMPRSS2* in SARS-CoV-2-infected lungs and control lungs. (**A**) Expression of *TMPRSS2* in SARS-CoV-2-infected lungs and control lungs. (**B**) Expression of *TMPRSS2* in different cell types in SARS-CoV-2-infected lungs and control lungs. “**”, *p* value < 0.01.

Because SARS-CoV infection downregulates *TMPRSS2* expression in cultured cells [22], we investigated the changes in *TMPRSS2* expression in COVID-19-infected lungs. By analyzing the single-cell RNA-sequencing dataset (GSE171524) of COVID-19-infected lungs and control lungs [23], the expression levels of *TMPRSS2* in COVID-19-infected lungs were significantly reduced compared to the control lungs (Figure 7A). By further analyzing different cell types, we found that the expression levels of *TMPRSS2* in SARS-CoV-2-infected lungs were reduced in epithelial cells but significantly increased in myeloid cells compared to control lungs (Figure 7B). Consistent with these results in Figure 7A, analysis of the GSE152075 dataset indicates that nasopharyngeal swabs of SARS-CoV-2-infected patients show significantly lower *TMPRSS2* expression in virus-infected individuals compared to healthy individuals [24]. Moreover, the expression levels of *TMPRSS2* were very high in epithelial cells of both SARS-CoV-2-infected lungs and control lungs (Figure 7B).

## 3. Discussion 

Highly expressed viral entry factors may play critical roles in the invasion of SARS-CoV-2 [25,26,27,28]. A TMPRSS2-expressing cell line has been shown to have high susceptibility for SARS-CoV-2 invasion [29,30]. In the present study, we conducted expression, mutation, and prognostic analyses for the *TMPRSS2* gene in pan-cancers and in COVID-19-infected lung tissues. TMPRSS2 expression was highest in prostate cancer followed by urothelial cancer, renal cancer, and pancreatic cancer. In addition, TMPRSS2 expression was low in lung cancer, and was not detected in other cancer tissues, including breast cancer. IHC revealed weak to moderate membranous and/or granular cytoplasmic immunoreactivity in lung and breast cancer tissues, which was supported by a previous study reporting that prostate cancer patients have a high risk for SARS-CoV-2 infection compared to non-cancer patients [31]. A high expression of *TMPRSS2* was significantly associated with a short overall survival in BRCA, SARC, and UVM, while a low expression of *TMPRSS2* was significantly associated with a short overall survival in LUAD. These results demonstrate a role of TMPRSS2 in SARS-CoV-2 invasion, cancer susceptibility, and cancer severity in patients with PRCA, BRCA, SARC, and UVM. We further analyzed TMPRSS2 expression using a dataset containing COVID-19-infected lungs and control lungs, and we found that the levels of TMPRSS2 in COVID-19-infected lungs were significantly reduced compared to the control lungs. Considering that a low expression of *TMPRSS2* is significantly associated with a short overall survival in LUAD, low *TMPRSS2* expression may result in severity and death of LUAD cancer patients infected with COVID-19 or SARS-CoV-2.

Mutation analysis of the pan-cancers revealed that the *TMPRSS2* mutation frequency was significantly higher in PRAD and that the mutated *TMPRSS2* group was significantly associated with long overall, progression-free, disease-specific, and disease-free survival compared to the WT *TMPRSS2* group, demonstrating a loss of function roles for *TMPRSS2* mutations as prognostic markers in pan-cancers. However, it remains unknown whether *TMPRSS2* mutation affects COVID-19 severity, thereby indicating the need for additional studies to understand the causation roles of *TMPRSS2* mutation in COVID-19.

Targeting SARS-CoV-2 entry factors, including TMPRSS2, may be a therapeutic strategy against COVID-19 [32,33,34]. Molecular docking analysis has predicted that small molecules have a functional inhibitory effect on TMPRSS2 [21]. Many dietary flavonoids show promising multitarget activities against SARS-CoV-2 [35]. A small-molecule compound, N-0385, has recently been reported to act as a pan-SARS-CoV-2 prophylactic and therapeutic agent with TMPRSS2 inhibitory activity [36]. Thus, the present study investigated whether small molecules (CD, AD, TQ, and TQFL12) regulate TMPRSS2 expression. We found that CD inhibited TMPRSS2 expression in H460, MCF7, MDA-MB-231, and PC3 cells. Moreover, we found that TQ inhibited TMPRSS2 expression in MCF7 and 22RV1 cells and that TQFL12 inhibited TMPRSS2 expression in PC3 and 22RV1 cells. Further, AD inhibited TMPRSS2 expression in H460 and 22RV1 cells. Notably, AD is a natural nucleotide from an intermediate product of metabolism in the human body, and has been reported to play roles in COVID-19 pathogenesis and therapeutic opportunities [37]. Moreover, CD treatment inhibited the translation and promoted the degradation of TMPRSS2. CD, TQ, and TQFL12 have anti-cancer suppressive roles both in vitro and in vivo [38,39,40,41]. Natural product CD is a derivative (analog) from AD, while TQFL12 is a novel synthetic derivative from TQ [38,42]. Taken together, these results imply that CD, AD, TQ, and TQFL12 may have therapeutic roles in preventing COVID-19 and cancers.

## 4. Materials and Methods

### 4.1. Expression Analysis in Databases

The human *TMPRSS2* gene expression levels in cancers were evaluated in The Cancer Genome Atlas (TCGA) database (accessed on date for this link, e.g., https://www.proteinatlas.org/ENSG00000184012-TMPRSS2/pathology, accessed on 1 September 2022), and the correlation of *TMPRSS2* gene expression levels with cancer survival was evaluated by Gene Expression Profiling Interactive Analysis (GEPIA 2, http://gepia2.cancer-pku.cn/#analysis, accessed on 1 September 2022) [43,44,45]. Mutation and survival analyses for TMPRSS2 in pan-cancers were performed using cBioPortal (https://www.cbioportal.org/results/cancerTypesSumary?case_set_id=all&gene_list=TMPRSS2&cancer_study_list=5c8a7d55e4b046111fee2296, accessed on 1 September 2022) in TCGA [46,47]. 

### 4.2. Reagents, Antibodies, and Cell Lines 

CD and TQ have been previously described [48], and TQFL12 is a new synthetic TQ-derivative [38]. AD was purchased from Macklin Inc. (Shangai, China, A6218-25 g). The TMPRSS2 antibody for Western blotting and immunohistochemistry (IHC) was purchased from Sigma-Aldrich (cat #: HPA035787, Burlington, MA, USA). The indicated cancer cell lines and culture conditions have been previously described [47,48].

### 4.3. Immunohistochemistry (IHC)

The IHC protocol has been previously described [47,48]. In brief, formalin-fixed paraffin-embedded tissues from Chinese breast cancer and lung cancer patients were subjected to antibody staining with the TMPRSS2 antibody (1:100 dilution) for IHC. Informed consent forms were obtained for the cancer patient tissues [47].

### 4.4. Western Blotting 

The PC3 and 22RV1 prostate cancer cell lines, the H460 lung cancer cell line, and the MCF7 breast cancer cell line were utilized in the present study. Western blotting for TMPRSS2 was performed using cells treated with or without CD, AD (0, 10, 20, and 40 µm), TQ, or TQFL12 (0, 5, 10, and 20 µm) for 24 h. β-actin antibodies were used as the internal controls. Assays for cycloheximide (CHX)-based protein stability were performed as previously described [49]. The 22RV1 cells were treated with and without CD and with indicated CHX, followed by Western blotting. All experiments were repeated three times.

### 4.5. Semi-Quantitative RT-PCR for TMPRSS2

The semi-quantitative RT-PCR assays were conducted using the above-described treated cancer cells. The following primers for RT-PCR were used: RT-TMPRSS2-L, 5′-caccaccagctattggacct-3′; and RT-TMPRSS2-R, 5′-acacgccatcacaccagtta-3′. The PCR product size was 390 bp. The *ACTB* and *GDPDH* genes were used as the internal controls. All experiments were repeated three times.

## 5. Conclusions

The *TMPRSS2* gene is highly expressed in cancer tissues, specifically in PRAD tumors, implying susceptibility to SARS-CoV-2 and severity for COVID-19. TMPRSS2 mutations may be a prognostic marker for long survival rates in pan-cancers. This is the first study to demonstrate that the small molecules CD, AD, TQ, and TQFL12 inhibit TMPRSS2 expression, which may have therapeutic roles via targeting TMPRSS2 to prevent SARS-CoV-2 invasion in cancer patients during the COVID-19 pandemic.

## Figures and Tables

**Table 1:** Survival of cancer patients with *TMPRSS2* mutations.

Survival Type	No. Patients	*p*-Value	*q*-Value
Overall	10,803	8.71 × 10^−11^	3.49 × 10^−10^
Disease-specific	10,258	5.66 × 10^−9^	1.13 × 10^−8^
Progression-free	10,613	1.17 × 10^−7^	1.56 × 10^−7^
Disease-free	5383	1.24 × 10^−5^	1.24 × 10^−5^

## Data Availability

Not applicable.
